# Viscoelastic and self-healing behavior of silica filled ionically modified poly(isobutylene-*co*-isoprene) rubber†

**DOI:** 10.1039/c8ra04631j

**Published:** 2018-07-27

**Authors:** Aladdin Sallat, Amit Das, Jana Schaber, Ulrich Scheler, Eshwaran S. Bhagavatheswaran, Klaus W. Stöckelhuber, Gert Heinrich, Brigitte Voit, Frank Böhme

**Affiliations:** Leibniz Institut für Polymerforschung Dresden, Hohe Straße 6 D-01062 Dresden Germany boehme@ipfdd.de; Organische Chemie der Polymere, Technische Universität Dresden D-01062 Dresden Germany; Institut für Textilmaschinen und Textile Hochleistungswerkstofftechnik, Technische Universität Dresden D-01069 Dresden Germany

## Abstract

Rubber composites were prepared by mixing bromobutyl rubber (BIIR) with silica particles in the presence of 1-butylimidazole. In addition to pristine (precipitated) silica, silanized particles with aliphatic or imidazolium functional groups, respectively, were used as filler. The silanization was carried out either separately or *in situ* during compounding. The silanized particles were characterized by TGA, ^1^H–^29^Si cross polarization (CP)/MAS NMR, and Zeta potential measurements. During compounding, the bromine groups of BIIR were converted with 1-butylimidazole to ionic imidazolium groups which formed a dynamic network by ionic association. Based on DMA temperature and strain sweep measurements as well as cyclic tensile tests and stress–strain measurements it could be concluded that interactions between the ionic groups and interactions with the functional groups of the silica particles strongly influence the mechanical and viscoelastic behavior of the composites. A particularly pronounced reinforcing effect was observed for the composite with pristine silica, which was attributed to acid–base interactions between the silanol and imidazolium groups. In composites with alkyl or imidazolium functionalized silica particles, the interactions between the filler and the rubber matrix form dynamic networks with pronounced self-healing behavior and excellent tensile strength values of up to 19 MPa. This new approach in utilizing filler–matrix interactions in the formation of dynamic networks opens up new avenues in designing new kinds of particle-reinforced self-healing elastomeric materials with high technological relevance.

## Abstract 2

A self-healing rubber/silica composite with superior mechanical performance was obtained by tuning specific noncovalent filler–matrix interactions.

## Graphical abstract

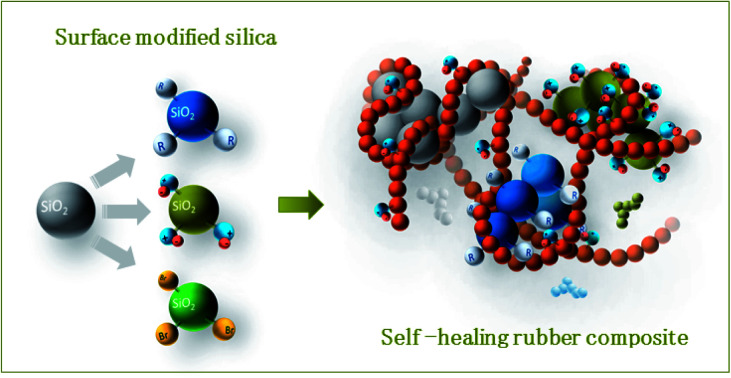

## Introduction

After the first description of a self-healing rubber by Leibler *et al.*,^1^ a series of publications appeared which aimed to implement self-healing behavior in commercial rubbers. This included materials like natural rubber,^2–4^ chloroprene rubber,^5^ polybutadiene,^6^ acrylonitrile butadiene rubber,^7^ styrene butadiene rubber,^8^ and polydimethylsiloxane.^9,10^ In order to facilitate self-healing, both physical^6–9^ and reversible covalent cross-linking^2–5^ have been utilized in these rubber systems, with strong focus on the basic principles of self-healing. In recent publications, the self-healing behavior of rubber composites has met increasing interest in materials research. Here, emphasis is put on carbon mixtures, the electrical conductivity of which is used for sensoric and electronic applications^11–13^ or Joule heating induced self-healing.^14^ In another example, IR laser induced self-healing was described for a graphene containing polyurethane rubber applicable in flexible electronics.^15^ A further example is a mixture with metallic fibers in which self-healing was pursued by microwave heating.^16^ However, the influence of reinforcing fillers on the self-healing behavior of rubber composites has received little attention so far.

Generally, compounding of technical rubbers with special fillers is a necessary measure to adapt material properties for respective technical applications, *e.g.* in tire components. This also applies to self-healing rubbers. The extent to which the additional interactions of the polymer matrix with the filler influence material properties of self-healing rubbers has not been sufficiently investigated yet.

**Scheme 1:**
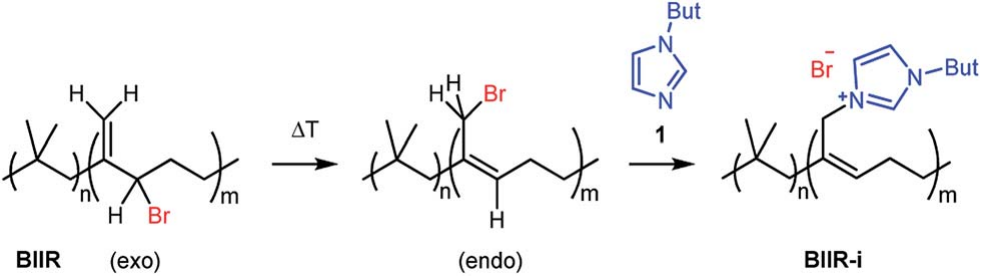
Ionic modification of bromobutyl rubber by conversion with 1-butylimidazole.

In this publication, we describe silica-rubber composites, the properties of which were adjusted *via* matrix–filler interactions. Ionically modified bromobutyl rubber (BIIR-i), which served as the rubber matrix, was prepared by conversion of BIIR with 1-butylimidazole (1) (see Scheme 1). This kind of modification was first described by Parent *et al.*^17,18^ In our own work, we have recently demonstrated that such modified rubbers show a pronounced tendency to self-heal.^19,20^ This self-healing effect was attributed to the formation and rearrangement of ionic clusters. Introduction of carbon nanotubes into BIIR-i facilitated self-healing by Joule heating.^21,22^ The aim of the present work was to determine to which extent the properties of BIIR can be further improved by the addition of silica without sacrificing self-healing. Our investigations show the general possibility of implementing self-healing behavior in technically relevant rubber formulations through ionic interactions. This approach is not limited to BIIR but might also be useful for other technical rubbers. Due to the self-healing behavior, it is expected that micro cracks formed during use will be repaired immediately, resulting in longer lifespans and preventing total material failure.^19^

**Scheme 2:**
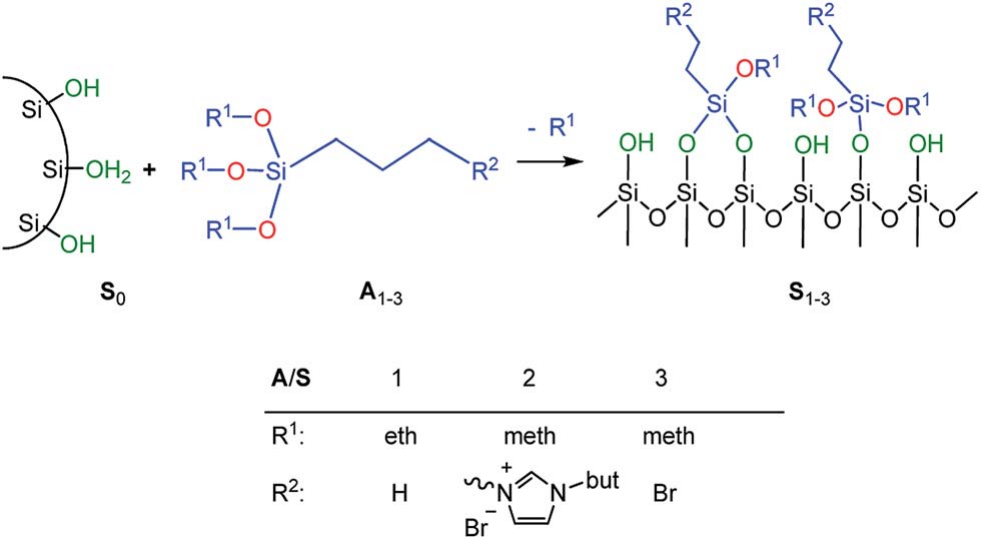
Silanization of precipitated silica (Ultrasil 7000GR).

In order to adjust the interactions between the rubber matrix and the filler, the filler surface was modified by silanization with three alkoxysilanes as shown in Scheme 2. Here, two different approaches were followed. In the first approach, the silica particles were silanized separately (*ex situ*) and then mixed with the rubber. In the second approach, the silanization was performed during mixing (*in situ*). The influence of different alkoxysilanes and mixing procedures on the material properties is discussed.

## Experimental

### Materials

1-Butylimidazole (1) (Sigma-Aldrich, 98%), *n*-propyltriethoxysilane (A_1_) (abcr, 97%), (3-bromopropyl)trimethoxysilane (A_3_) (abcr, 97%), toluene (Aldrich, anhydrous 99.8%), and methanol (Acros Organics, 99.9%) were used as received. Precipitated silica Ultrasil® 7000 GR (S_0_) with a specific surface area (BET) of 175 m^2^ g^−1^ and a primary particle size of 10 nm was supplied by Evonik Industries. Bromobutyl rubber (BIIR) is a commercial product of Lanxess with a bromine content of 1.13 wt% (0.80 mol% brominated isoprene units) determined by ^1^H NMR. Ionically modified bromobutyl rubber (BIIR-i) was obtained by conversion of BIIR with 1 (see Scheme 1) as described earlier.^19,20^

### 1-Butyl-3-(trimethoxysilylpropyl)imidazolium bromide (A_2_)

A mixture of (3-bromopropyl)trimethoxysilane (35 mL, 267 mmol) and 1-butylimidazole (50 mL, 267 mmol) was stirred in a dried round bottom flask for 5 days at room temperature. A_2_ was obtained as yellow viscous oil which was used without further purification.

^1^H NMR (CDCl_3_ ppm): *δ*_H_ = 10.57 (1H, s, Im–H^9^) 7.46 (1H, s, Im–H^10 alt 11^) 7.39 (1H, s, Im–H^10 alt 11^), 4.36 (4H, m, H^4,5^), 3.55 (9H, s, H^1^), 2.01 (2H, t, H^3 alt 6^), 1.91 (2H, t, H^3 alt 6^), 1.38 (2H, m, H^7^), 0.95 (3H, t, H^8^), 0.63 (2H, t, H^2^).
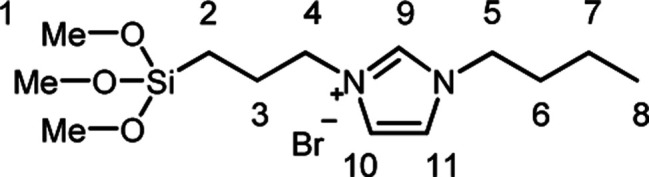


### Silanization of silica particles (S_1–3_)

Three different types of surface-modified silica particles were obtained by silanization of S_0_ with alkoxysilanes such as *n*-propyltriethoxysilane (A_1_), 1-butyl-3-(trimethoxysilylpropyl)imidazolium bromide (A_2_), and (3-bromopropyl)trimethoxy-silane (A_3_) respectively. The silanization was performed as follows: an amount of 15 g Ultrasil® 7000 GR were suspended in 250 mL of dry toluene in a dried 500 mL round bottom flask equipped with a reflux condenser. An excess of the alkoxysilane (2.8 mmol g^−1^ silica) was added while stirring. The suspension was heated under reflux for 24 h. Then, methanol formed during the reaction was distilled off. After cooling, the modified silica particles were collected by centrifugation and thoroughly washed with methanol. The product was then dried at 110 °C for 12 h.

### Preparation of silica-rubber composites (C_0–5_)

An amount of 50 g BIIR (6.94 mmol allylic bromide) and 1.41 g of 1-butylimidazole (11 mmol) were premixed in an internal mixer (Haake Rheomix, Thermo Electron GmbH, Karlsruhe, Germany) for 10 min with a rotor speed of 60 rpm at 40 °C. Under these conditions, grafting reactions according to Scheme 1 can be neglected. This mixture was used as a master batch for the preparation of composites C_0–5_. Two different methods were used for the preparation of the composites.

#### *Ex situ* silanization approach (C_1–3_)

A portion of the BIIR/1-butylimidazole master batch was mixed with 30 phr of the pre-silanized silica particles S_1–3_ in an internal mixer with a rotor speed of 50 rpm at 40 °C for 10 min. After that, the compounded mass was homogenized in a laboratory size two-roll mixing mill (Polymix 110L, size 203 × 102 mm, Servitech GmbH, Wustermark, Germany) at 50 °C for 2 min and then molded under pressure at 120 °C for 30 min. Using the same method, a reference sample (C_0_) was prepared by mixing the master batch with pristine silica S_0_.

#### *In situ* silanization approach (C_4–5_)

A portion of the BIIR/1-butylimidazole master batch was mixed with 30 phr of pristine silica S_0_ in an internal mixer with a rotor speed of 50 rpm at 110 °C for 2 min. Then, an amount of 2.5 phr of the silane coupling agent (A_1_, A_3_) was added and allowed to react with the silica for 5 min. During that time, the temperature raised rapidly to 140 °C. After that, the compounded mass was homogenized in a laboratory size two-roll mixing mill at 50 °C for two min and then molded under pressure at 120 °C for 30 min. The amount of added silane coupling agent was calculated as described by Mihara *et al.*^23^

In both cases, the formation of BIIR-i according to Scheme 1 occurred *in situ* during processing at temperatures higher than 100 °C. A sample overview is given in Table 1. After molding, test bars of the composites were punched out of the sheet obtained and used for mechanical and self-healing tests. The transparency of all samples indicates a homogeneous particle distribution. This is confirmed by TEM images of selected samples (see ESI SI1 and SI2†).

**Table 1:** Sample overview

Sample	BIIR [phr]	Comp. 1 [phr]	Silica (S) [phr]	Alkoxysilane (A) [phr]	Functional groups^e^
BIIR-i	100	3	—	—	—
C_0_^a^	100	3	30 (S_0_)	—	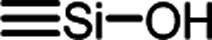
C_1_^a^^,^^b^	100	3	30 (S_1_)	—	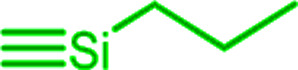
C_2_^a^^,^^b^	100	3	30 (S_2_)	—	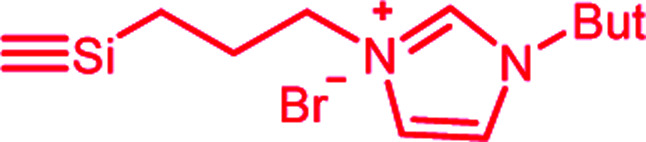
C_3_^b^^,^^c^	100	3	30 (S_3_)	—	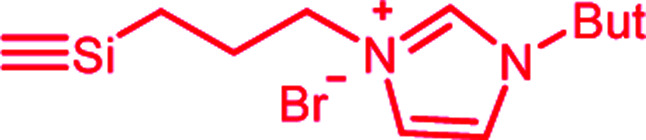
C_4_^c^^,^^d^	100	3	30 (S_0_)	2.5 (A_1_)	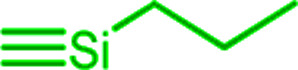
C_5_^c^^,^^d^	100	3	30 (S_0_)	2.5 (A_3_)	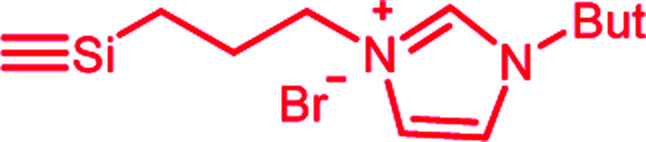

a No change in surface functionality during compounding.

b *Ex situ* particle silanization approach.

c Change in surface functionality during compounding.

d *In situ* particle silanization approach.

e Functional groups on the particle surface after compounding.

### Characterization

^1^H NMR (500.13 MHz) spectra were recorded on an Avance III 500 NMR spectrometer (Bruker). CDCl_3_ (*δ*(^1^H) = 7.26 ppm) was used as the solvent and internal standard.

^1^H–^29^Si cross polarization (CP)/MAS NMR spectra were recorded using an Avance III 300 MHz spectrometer (Bruker, Karlsruhe, Germany) with a double resonance HX 4 mm MAS probe head as described by Fischer *et al.*^24^ Q8M8 (*δ*(Si(–CH_3_)_3_) = 12.6 ppm) was used as reference for ^29^Si. The CP/MAS NMR experiments were carried out with a π/2 pulse duration of 4 μs for ^1^H, a contact time *τ* of 2 ms and 20 000 scans at a MAS spinning rate of 10 kHz. One pulse 1H MAS NMR experiments were also carried out using a π/2 pulse duration of 4 μs and 10 scans at a MAS spinning rate of 10 kHz.

Thermogravimetric analysis was performed using a TGA Q 5000 (TA instruments, New Castle, DE, USA) with a heating rate of 10 K min^−1^ under nitrogen atmosphere. The weight loss from 50 to 800 °C was measured.

Zeta potential measurements were performed on pristine (S_0_) and modified silica particles (S_1–3_) using a Zetasizer Nano (Malvern Instruments Inc., Malvern, UK). The determination of the Zeta potential was based on the electrophoretic mobility measured at a voltage of 40 V and an electrode distance of 5 cm. The samples (each 30 mg) were dispersed in 30 mL of an aqueous solution of KCl (*c* = 10^−3^ mol L^−1^) in an ultrasonic bath. The pH was adjusted with HCl and KOH (*c* = 0.1 mol L^−1^). With the measured electrophoretic mobility

1 *μ* = *ν*/*E*,

the Zeta potential *ζ* was calculated using the simple Smoluchowski equation:2
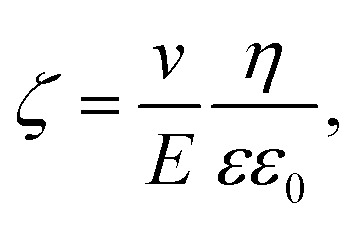
where *ν* is the drift velocity, *η* is the viscosity, *E* is the electric field strength, *ε* is the dielectric constant, and *ε*_0_ is the vacuum permittivity.

Dynamic mechanical analysis (DMA) was performed on standard test bars (5 × 10 × 2 mm) with a thermal spectrometer (EPLEXOR 2000N) from GABO QUALIMETER, Ahlden, Germany. The temperature sweep measurements were carried out in tensile mode and in a temperature range from −80 to +80 °C with a heating rate of 2 K min^−1^ at a frequency of 10 Hz under 0.5% (dynamic) and 1% (static) strain. The strain sweep measurements were performed at a constant frequency of 10 Hz with 60% pre-strain and dynamic strain from 0.01–30%.

Tensile tests were carried out on a Zwick 1456 universal testing machine at a constant stretch rate of 200 mm min^−1^ according to DIN EN ISO 527-2/S2/20.

Self-healing tests were performed as described earlier.^20^ For this, test bars of the composites were placed in a custom-built test device, cut with a razor blade and then pressed together with a defined compression of 0.2 mm. In this state, the samples were allowed to heal for 16 h at 70 °C and then stored at room temperature. Finally, the mended samples were subjected to tensile tests. The tensile stress (*σ*_b_)- and elongation at break (*ε*_b_)-related healing efficiencies *H*_σ_ and *H*_ε_ (in %) were calculated from the ratios of the respective parameters of the virgin and the healed samples.

## Results and discussion

### Surface-modified silica particles

The preparation of suitable composites based on silica and ionically modified rubber (BIIR-i) requires an adjustment of the interactions between the silica particles and the matrix. For this, the surface of silica (Ultrasil® 7000 GR) was silanized according to Scheme 2 using three different alkoxysilanes (A_1–3_). The modification with A_1_ (S_1_) aimed to adapt the interactions of the particles with the hydrophobic backbone of BIIR-i, whereas with A_2_ (S_2_), an improvement of the interactions with the ionic part of BIIR-i was envisioned. Surface modification with A_3_ provided silica particles with reactive bromine groups at the surface (S_3_) to be used for the *in situ* formation of ionic groups on the silica surface during mixing with BIIR/1-butylimidazole.

**Fig. 1:**
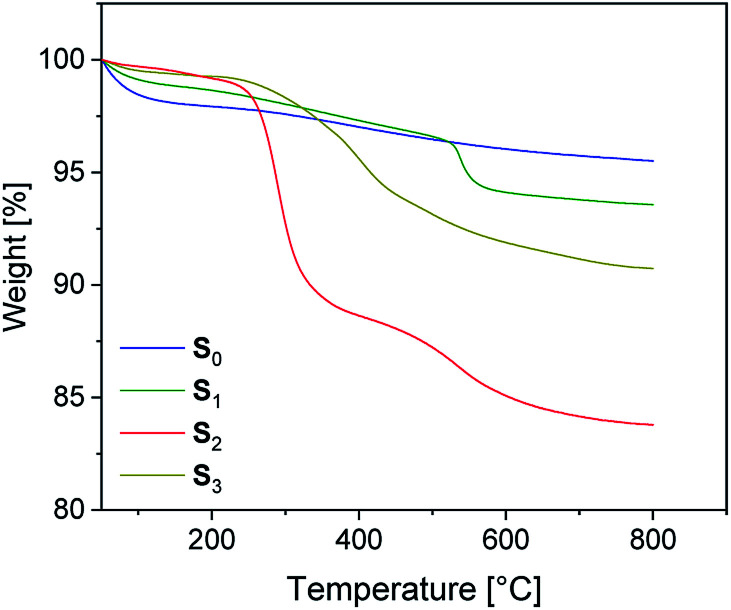
TGA traces for unmodified silica S_0_ and surface-modified silicas S_1–3_.

Pristine silica S_0_ and the pre-silanized (*ex situ*) silicas S_1–3_ were characterized by TGA in the temperature range from 20 to 800 °C. According to the thermograms shown in Fig. 1, all samples revealed an initial weight loss at temperatures up to 100 °C. This is due to the removal of physically adsorbed water and any solvent residues remaining from the modification process. Up to 200 °C the physically adsorbed water is completely removed. In the region from 200 to 800 °C, a gradual weight loss is observed for the unmodified silica S_0_ (Fig. 1), which is attributed to dehydroxylation reactions, in which silanol groups condense to siloxane bridges.^25^

The alkyl modified silica S_1_ exhibits gradual thermal degradation from 200 to 520 °C and a pronounced degradation step at 540 °C. For the bromopropyl modified sample S_3_, a very broad degradation step above 200 °C is found, whereas the ionically modified S_2_ exhibits two pronounced degradation steps at about 270 and 550 °C. It is assumed that the degradation behavior of the modified samples S_1–3_ is superimposed by three different degradation processes. The first one is the dehydroxylation of remaining silanol groups as already discussed for S_0_. The others are the decomposition of the functional groups (Br and imidazolium, respectively) and of the surface-bonded residual alkoxy side groups.^26^ The TGA curve of S_1_ suggests that the latter decompose at higher temperatures. The very broad degradation step of S_3_ above 200 °C is assumed to be the result of a superposition of debromination and decomposition of the alkoxy side groups.

The weight loss of the alkoxysilane modified silica particles above 200 °C is compared to that of unmodified silica. The results are listed in Table 2. From the values of unmodified silica we can conclude that the dehydroxylation process on the surface accounts for a 2% weight loss. The modified silicas S_1_ and S_3_ have a total weight loss of 4.7 and 8.1% respectively, while the modified silica S_2_ has a total weight loss of 15%.

**Table 2:** Thermogravimetric weight loss of unmodified-(S_0_) and modified silica (S_1_–S_3_)

Temperature [°C]	S_0_ [%]	S_1_ [%]	S_2_ [%]	S_3_ [%]
200–400	0.9	1.2	10.5	3.7
400–700	1.3	3.5	4.5	4.5
200–700	2.2	4.7	15.0	8.1

**Fig. 2:**
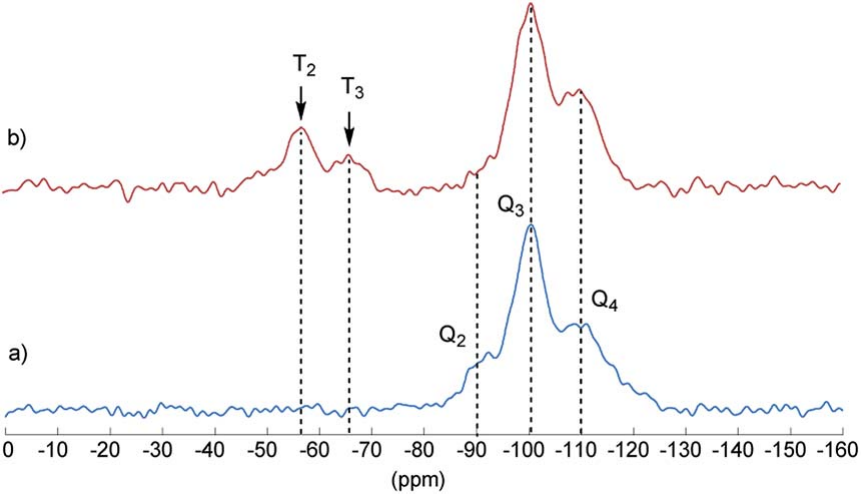
^1^H–^29^Si CP/MAS NMR spectrum of unmodified silica S_0_ (a) and modified silica S_2_ (b) with assignments of T and Q groups. Q_2_ = geminal silanol, Q_3_ = single silanol, Q_4_ = siloxane bridges. Contact time *τ* = 2 ms.

**Fig. 3:**
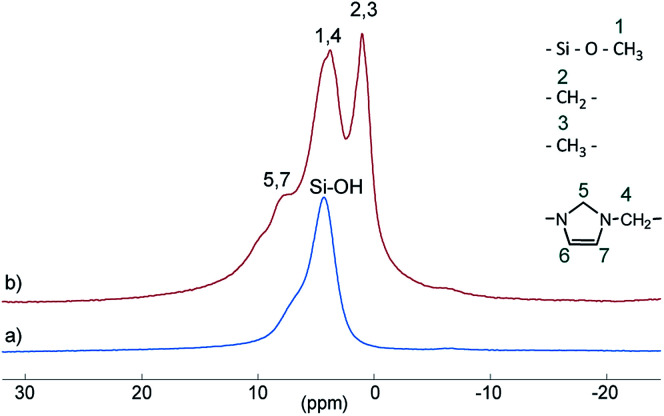
^1^H MAS NMR spectra of unmodified silica S_0_ (a) and modified silica S_2_ (b).

Final proof for the grafting of alkoxysilanes on the silica surface was obtained by ^29^Si CP/MAS NMR spectroscopy as previously described.^24^ These investigations were carried out with S_2_ as an example. Fig. 2 shows the ^29^Si CP/MAS NMR spectra of S_0_ and S_2_. In the spectrum of S_0_, the typical Q_2_, Q_3_, and Q_4_ signals belonging to geminal (=Si–(OH)_2_) and vicinal silanol (≡Si–OH) groups as well as to siloxane bridges (Si–(O–Si)_4_), respectively, are visible.^27^ After conversion with A_2_, two new signals appeared at −57 (T_2_) and −66 ppm (T_3_), which are assigned to Si–O–SiR–(OMe)_2_ and (Si–O)_2_–SiR–OMe units, indicating the formation of covalent bonds between silica and the organic moieties.^25^ The R group here corresponds to the alkylsilane moiety. The geminal silanol (Q_2_) signal almost disappeared after modification of the silica surface with A_2_, indicating that most of the Q_2_ sites have reacted and the majority of the residual silanol groups are of Q_3_ type. Additionally, the grafted alkylsilane on the surface of S_2_ can be detected by ^1^H MAS NMR spectroscopy (see Fig. 3). The spectrum of the pristine silica S_0_ exhibits a broad signal at about 4.4 ppm which is assigned to Si–OH groups. Additional signals in the spectrum of S_2_ are attributed to the imidazolium group (low field) and to the aliphatic groups of the alkyl chain (high field). The signals of unconverted alkoxysilane groups (Si–O–CH_3_) and the signals of the CH_2_ groups attached to the imidazolium moiety (N–CH_2_–) overlap with the Si–OH signal.

**Fig. 4:**
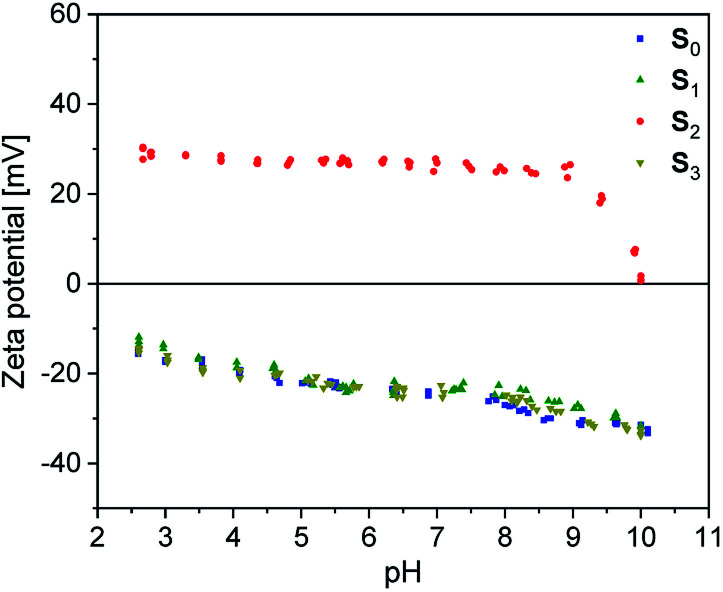
Zeta potential of unmodified (S_0_) and alkoxysilane modified silica fillers (S_1–3_) in dependence of pH.

In order to get information about the surface polarization of pristine and modified silica particles (S_1–3_), Zeta potential measurements were performed (see Fig. 4). The Zeta potential of pristine silica (S_0_) is essentially determined by the dissociation of the slightly acidic silanol groups on the particle surface (Si–OH ↔ Si–O^−^ + H^+^). The resulting negatively charged surface causes the negative Zeta potential determined over the whole pH range from 2.5 to 10. The isoelectric point (IEP) to be expected at lower pH values is outside the selected measuring range. The modification of S_0_ with the alkoxysilanes A_1_ and A_3_ does not lead to significant changes in the shape of the Zeta potential curves. Obviously, the surface charge of S_1_ and S_3_ is not strongly influenced by the modification. Here, it is assumed that the Zeta potential is mainly determined by remaining unreacted silanol groups whereas the influence of the grafted non-dissociable alkylsilane groups is negligible.

Completely different behavior is seen in S_2_, which was modified with A_2_. The cationic alkyl imidazolium groups at the surface of S_2_ result in a basic surface with a positive Zeta potential. Neutralization of the surface charge only occurs at the IEP at pH = 10. Evidently, the influence of the imidazolium groups distinctly overcompensates the influence of unreacted silanol groups.

### Rubber-silica composites

Composites C_0–3_ were prepared by mixing pristine silica (S_0_) or pre-silanized particles (S_1–3_) respectively with a master batch of BIIR and unreacted 1-butylimidazole (1) in an internal mixer at 40 °C (*ex situ* silanization approach). This procedure proved to be advantageous, since direct mixing of the silica particles with BIIR-i was difficult because of the higher viscosity of BIIR-i compared to unmodified BIIR.

For comparison, the silanization of the silica particles was also performed during compounding (*in situ* silanization approach, C_4–5_). For this, the master batch was consecutively mixed at 110 °C with pristine silica S_0_ and the alkoxysilanes A_1_ and A_3_ respectively. Due to the high shear forces, the temperature rose to *ca.* 140 °C, which is nearly the optimal temperature for the *in situ* silanization reaction. It is assumed that the reaction with A_1_ leads directly to the formation of S_1_ (C_4_), while in the reaction with A_3_ initially forms S_3_ which subsequently converts to S_2_ by reaction of the bromine group with 1 (C_5_).

Finally, all mixtures (C_0–5_) were homogenized in a two-roll mixing mill and then molded at 120 °C for 30 minutes. Based on our previous results,^19,20^ it is assumed that during this procedure BIIR-i is formed quantitatively by conversion of the polymer bound bromine groups with 1 according to Scheme 1. This reaction starts at temperatures above 50 °C and is assumed to be completed during molding. After molding, test bars of the composites were punched out and used for mechanical and self-healing tests.

Owing to the specific kinds of functional groups on the surface of S_0–2_ (silanol, alkyl, imidazolium), composites C_0–2_ presumably do not undergo changes in their filler surface functionality during compounding. Therefore, clearer correlations of their structure–property relationships are expected. In contrast, chemical reactions should occur on the filler surface during the preparation of C_3–5_, as is intended. For these samples, the results have to be regarded critically, since the extent of reactions at the particle surface cannot be accurately determined. Because of their presumably more defined structure, the focus of the following discussion is mainly placed on samples C_0–2_. Results concerning the reactive systems C_3–5_ are documented in the ESI (SI3–SI7†) and discussed comparatively at the end.

**Fig. 5:**
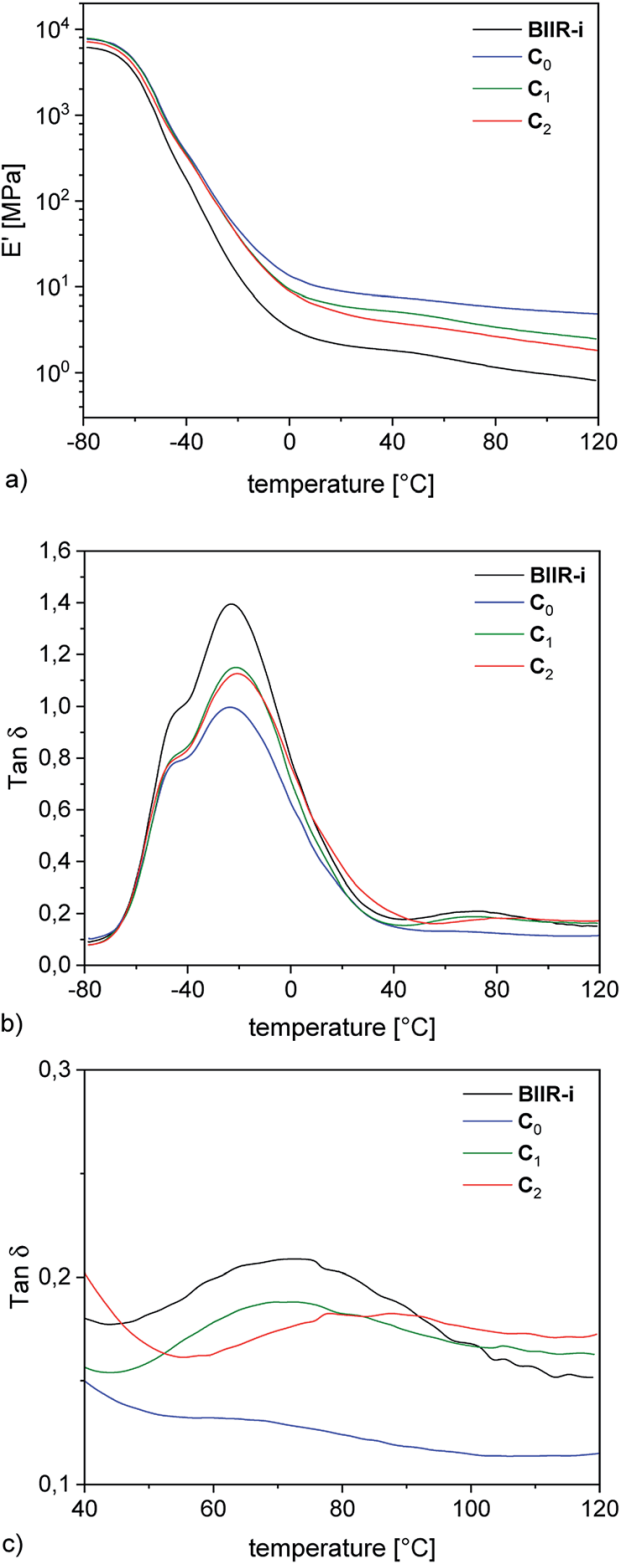
DMA temperature sweep measurements of rubber-silica composites C_0–2_ and BIIR-i (a) storage modulus plots (b) tan *δ* plots (c) enlarged section of the tan *δ* plots.

**Fig. 6:**
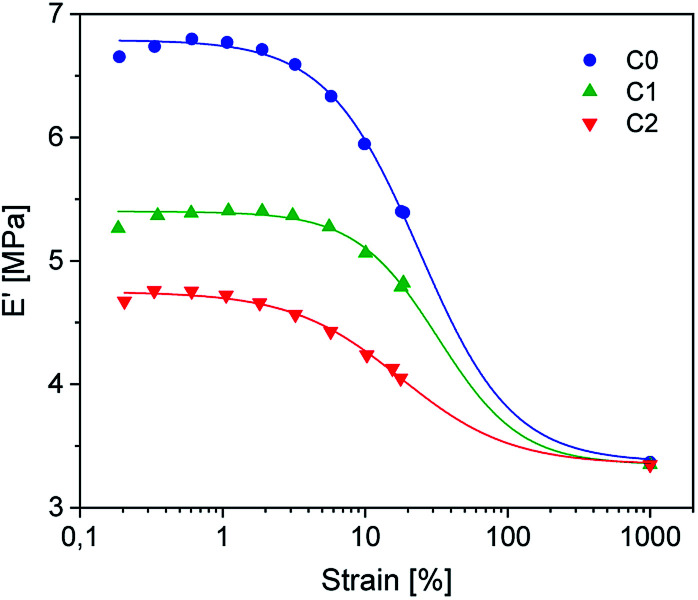
DMA temperature strain sweep measurements of rubber-silica composites C_0–2_. The symbols represent the measured variables. The lines are fitted according to the Kraus model.

**Fig. 7:**
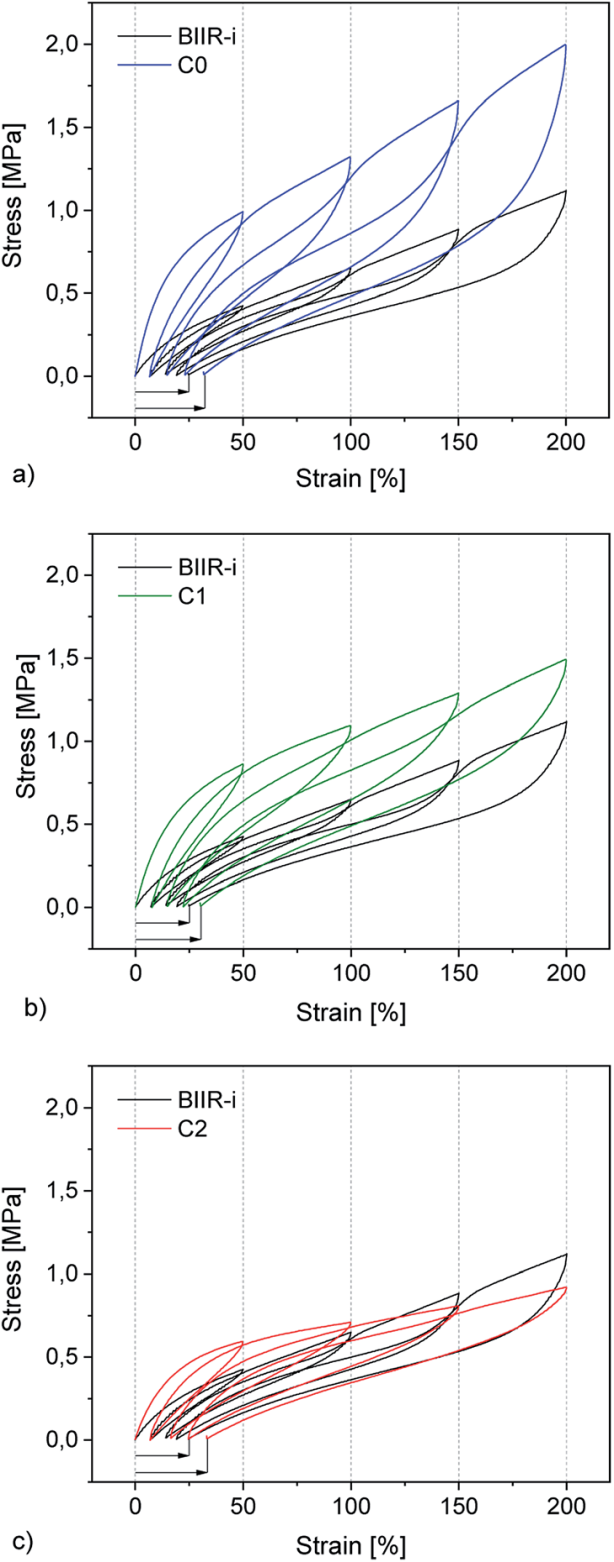
Mechanical hysteresis curves of (a) C_0_, (b) C_1_, and (c) C_0_ in comparison to BIIR-i.

**Fig. 8:**
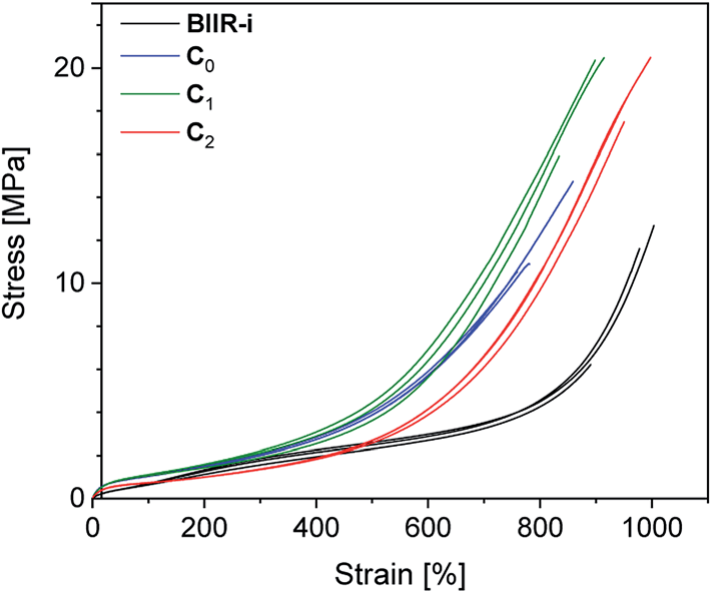
Stress–strain curves of composites C_0–2_ compared to BIIR-i.

Structurally, three different kinds of composites were obtained, distinguished by the functional groups present on the surface of their fillers (see Table 1). In the following (Fig. 5–8) and in the ESI (SI3–SI7†), the color of the curves indicates the specific functional groups. Blue stands for silanol groups (C_0_), green for alkyl groups (C_1_, C_4_), red for ionic imidazolium groups (C_2–3_, C_5_) and black for BIIR-i.

### Dynamic mechanical analysis

The DMA temperature sweeps of C_0–2_ and pure BIIR-i are depicted in Fig. 5. The storage modulus curves in Fig. 5a show a strong filler influence on the dynamic behavior of the composites. Compared to BIIR-i, the composites exhibit distinctly increased storage moduli in the application relevant temperature range. This effect is most pronounced in the composite with pristine silica (C_0_). For composites with modified silica (C_1–2_), the reinforcing effect is somewhat weaker, with slightly better values for the sample with alkyl functional groups on the filler surface (C_1_).

Regarding the reinforcing effect, the tan *δ* curves shown in Fig. 5b reveal a similar tendency. Generally, all samples exhibit the typical broad relaxation at the glass transition of BIIR with the maximum at about −22 °C. This relaxation is particularly pronounced for BIIR-i and decreases strongly in C_0_. The samples with modified silica (C_1–2_) are somewhere in between. Both the storage moduli and the low temperature relaxation are indicators of the prevailing network density in the composites, which is relatively small in BIIR-i and becomes more pronounced upon compounding with silica.

The dynamic behavior of the composites correlates well with the surface properties of the filler regardless of whether silanization was performed *in situ* or *ex situ*. Zeta potential measurements revealed an acidic surface of pristine silica which obviously causes increased interactions with the basic imidazolium groups of BIIR-i in C_0_ resulting in reduced chain mobility within the composite network. Introduction of flexible alkyl groups (C_1_) does not significantly influence the surface polarity of the fillers, but leads to shielding effects which reduce interactions between the filler and the matrix slightly. In the composite with the imidazolium modified filler (C_2_), ionic interactions between the components are assumed which, however, do not exceed the effect of acid–base interactions in C_0_.

In the tan *δ* curves, a further small relaxation at temperatures above 40 °C is visible (see Fig. 5c), which is attributed to reversible network formation processes in BIIR-i.^20^ Again, this relaxation is only weakly pronounced in C_0_ due to the acid–base interactions between the rubber matrix and the filler. In comparison to BIIR-i, this relaxation is slightly reduced in C_1_ due to the shielding effect of the aliphatic groups. In C_2_, this relaxation is shifted to higher temperatures. For this sample, the formation of larger ionic associates is assumed, whose complete dissolution requires higher temperatures.

For the samples with undefined filler surface functionalization (C_3–5_, see ESI SI3c†) the relaxation behavior in this temperature range is not completely clear. However, this relaxation tends to appear at lower temperature for composites with aliphatic substituents on the filler surface. This is an additional indication of the assumed shielding effect of aliphatic substituents.

An essential structural influence on the properties of composites lies in the distribution of the fillers. Competing interactions of the filler particles with each other or with the matrix lead to the formation of particle clusters or filler–filler networks which are susceptible to external mechanical forces.

Stress induced re-agglomeration processes usually referred to as “filler flocculation” can be investigated by dynamic mechanical amplitude sweep measurements. Such investigations give hints about the stability of these filler–filler networks and their influence on the mechanical performance of composite materials. Changes in the storage and loss modulus with increasing strain, well known as the Payne effect,^28^ can be attributed to a break-down of the filler network and to an increased energy dissipation during dynamic mechanical load.

Dynamic mechanical strain sweep measurements of C_0–2_ are shown in Fig. 6 (for C_3–5_ see ESI SI4†).

The data fit (solid lines) was performed according to the Kraus model (eqn (4))^29^ under consideration of the hydrodynamic reinforcement values (
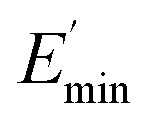
 at 1000% strain) extrapolated using the Chen and Acrivos approach (eqn (3))^30,31^ where the value *b* is assumed to be 5.01.3
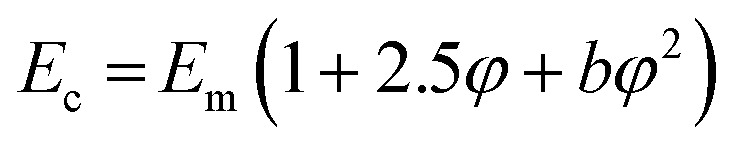
4
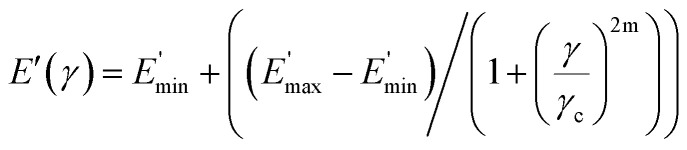


*E*_c_ and *E*_m_ are the dynamic elastic moduli of the composite and the pure matrix obtained from dynamic strain sweep measurements, *φ* is the volume fraction of silica (30 phr in the present study), *E*′(*γ*) is the storage modulus at a given dynamic strain, 
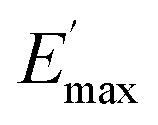
 is the storage modulus at very low dynamic strain, 
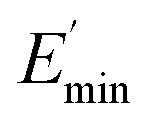
 represents a strain regime at which only hydrodynamic reinforcement effects exist^32^ and interactions between the particles become negligible, *γ* is the tensile strain amplitude, *γ*_c_ is the critical strain amplitude defining the point where 
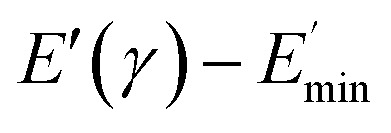
 becomes 50% of 
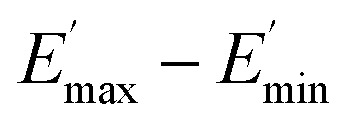
, *m* is a constant which is related to the specific fractal dimension of the filler clusters determining the shape of the curve.^33^

The extrapolation of 
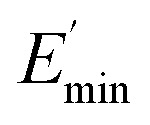
 using eqn (3) was necessary since measurements at high dynamic strain amplitudes are experimentally limited.^34^ The difference 
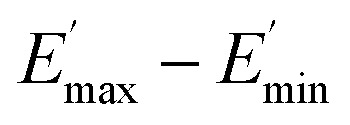
 can be regarded as a characteristic measure for the strength of the filler–filler network. The characteristic values of the Kraus model determined are summarized in Table 3.

**Table 3:** Fit parameters of the Kraus model for composites C_0–5_

Sample	Silica surface functional groups	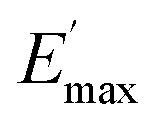 [MPa]	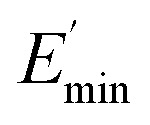 [MPa]	*γ*_c_	2*m*
C_0_^a^	Silanol	6.79	3.37	24.00 ± 1.04	1.33 ± 0.08
C_1_^a^^,^^b^	Alkyl	5.40	3.35	32.56 ± 1.51	1.51 ± 0.23
C_2_^a^^,^^b^	Imidazolium	4.75	3.35	17.77 ± 1.13	1.13 ± 0.08
C_3_^b^^,^^c^	Imidazolium	3.99	3.34	5.42 ± 1.73	1.73 ± 0.22
C_4_^c^^,^^d^	Alkyl	5.75	3.35	20.80 ± 1.54	1.48 ± 0.18
C_5_^c^^,^^d^	Imidazolium	4.84	3.36	10.15 ± 0.36	1.57 ± 0.09

a No change in surface functionality during compounding.

b *Ex situ* particle silanization approach.

c Change in surface functionality during compounding.

d *In situ* particle silanization approach.

Regarding the 
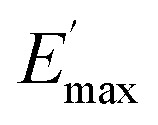
 values of C_0–2_, the trend is the same as found in the temperature sweep measurements. 
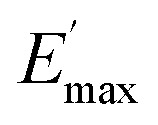
 decreases in the order C_0_ > C_1_ > C_2_. A slightly different tendency is seen for *γ*_c_, the point at which 50% of the filler–filler contacts are broken.^35^ Here, the order is C_1_ > C_0_ > C_2_. Actually, the stronger the filler network of the flocculated filler particles, the higher the Payne effect 
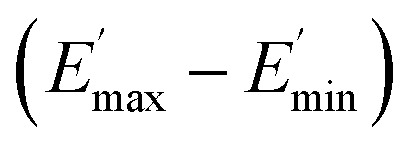
. In the case of C_0_, the flocculation tendency of the unmodified silica particles is strong, which leads to the most pronounced Payne effect. A modification of the filler surface by alkyl grafts (C_1_) reduces the difference in surface energies between filler and polymer matrix and leads to a reduced Payne effect as expected. In the case of C_2_, it is assumed that strong ionic interactions between the filler and the matrix prevent the formation of a strong filler network resulting in a lower critical strain. A very low value of *γ*_c_ is found in the case of C_3__,_ indicating that the filler flocculation in this sample is strongly suppressed. Here, the reaction of the bromine groups on the filler surface with imidazole takes place during molding. Because of the static molding conditions, the resulting ionic groups do not assist the dispersion. However, if the ionic groups are formed during mixing as in C_2_, the ionic interactions between silica and the rubber chains facilitate the dispersion of the silica.

### Tensile properties

Cyclic tensile tests and stress–strain measurements were performed on composites C_0–5_ and compared to BIIR-i. The mechanical hysteresis curves and stress–strain curves of BIIR-i and the rubber composites C_0–2_ are shown in Fig. 7 and 8 (for C_3–5__,_ see ESI SI5 and SI6†).

As can be seen from Fig. 7, all samples exhibit distinct strain softening (Mullin's effect) which can be attributed to irreversible rearrangements in the network. Due to the lack of a classical covalent network, permanent deformation occurs with increasing stress, resulting in increasing residual strain values after each cycle. The arrow lengths in Fig. 7 indicate the residual strain after four cycles. For the composites, these values are somewhat higher as for BIIR-i, showing that the incorporation of fillers results in higher levels of irreversible rearrangements during strain. On the other hand, the incorporation of fillers causes a significant reinforcing effect which can be seen in a steeper curve progression at low strain (up to 200%). This effect is strongest in the case of C_0_ and relatively weak for C_2_. This is in very good correlation with the DMA results and confirms our assumptions concerning the influence of the different filler surfaces. The enclosed area in the hysteresis loops is a measure of the energy dissipated in the material. The obtained results are in good agreement with the findings of the Payne effect measurements shown above. The reinforcing effect of the fillers clearly appears also at higher elongation (see Fig. 8). The stress–strain curves, measured three times for each sample, show a reasonably reproducible curve progression. All samples show pronounced strain hardening which, however, starts earlier in the composites compared to BIIR-i. Additionally, these measurements confirm the similarity of the mechanical behavior of C_2_ to that of unreinforced BIIR-i. Concerning the tensile strengths and elongation at break values (see Table 4), the influence of the filler type is not completely clear. We assume that the different filler types lead to different degrees of homogeneity of the filler distribution, which sensitively influence the ultimate rubber properties. Although this effect is important for technical rubber applications, it is not an issue for our fundamental study here. We have shown that an improvement of the tensile strengths by adding fillers tends to occur in all cases. Taking into account that no covalent crosslinking occurs, the mechanical behavior of all samples must be considered excellent. This applies especially for the samples C_1_ and C_2_, the filler surface of which was modified with aliphatic and ionic functional groups respectively.

**Table 4:** Tensile properties and healing efficiencies of composites C_0–5_ and BIIR-i

Sample	*σ*_b(v)_ a [MPa]	*ε*_b(v)_ b [%]	*H*_σ_ c [%]	*H*_ε_ d [%]
BIIR-i	10.2 ± 3.5	960 ± 60	87 ± 50	100 ± 11
C_0_	12.0 ± 2.3	800 ± 50	40 ± 25	55 ± 19
C_1_	18.9 ± 2.6	880 ± 40	43 ± 23	71 ± 12
C_2_	18.9 ± 1.5	970 ± 30	32 ± 16	72 ± 12
C_3_	10.1 ± 0.7	1030 ± 30	13 ± 8	41 ± 27
C_4_	11.7 ± 1.5	820 ± 30	n.d.^e^	n.d.^e^
C_5_	9.4 ± 0.5	790 ± 20	n.d.^e^	n.d.^e^

a Average tensile stress at break of the virgin samples.

b Average elongation at break of the virgin samples.

c Healing efficiency related to the tensile stress at break.

d Healing efficiency related to the elongation at break.

e Not determined.

### Self-healing behavior

**Fig. 9:**
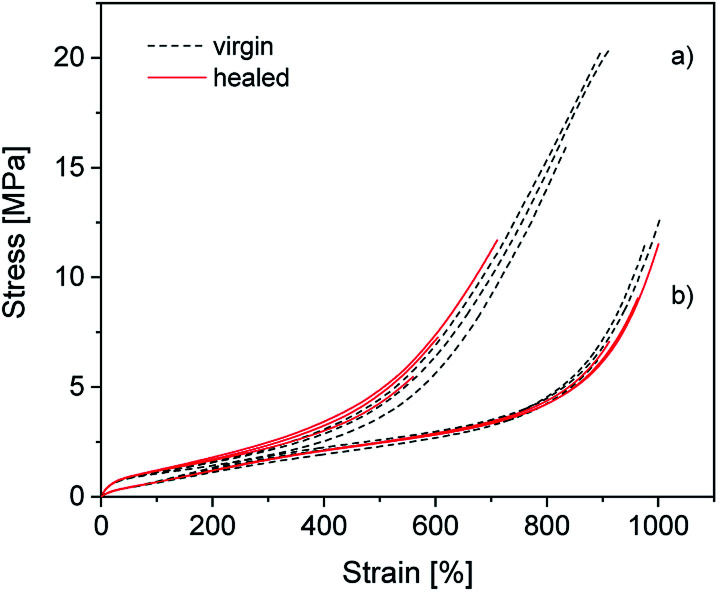
Stress–strain curves of (a) C_1_ and (b) BIIR-i. The black (dotted) and the red (solid) curves represent the virgin and the healed samples respectively.

To determine how compounding with silica affects the excellent self-healing behavior of ionically modified BIIR, self-healing tests were performed for C_0–3_ and compared to BIIR-i. For this, test bars of the samples were cut in the middle and then allowed to mend for 16 h at 70 °C under slight pressure. The mended samples were subjected to tensile tests. As an example, the self-healing effect is demonstrated for C_1_ and BIIR-i by means of stress–strain curves before and after healing (see Fig. 9). Respective plots for the other samples are shown in Fig. SI7 in the ESI.† Tensile stress and elongation at break related healing efficiencies *H*_σ_ and *H*_ε_ for all samples (mean values of three measurements) are summarized in Table 4. The results confirm the pronounced self-healing behavior of BIIR-i at reasonable overall performance, as already discussed earlier.^19,20^ The healing efficiencies of the composites are distinctly lower than for BIIR-i but still at a reasonable level. For the composites with surface-modified particles (C_1–2_) in particular, the stress at break values after healing (*σ*_b(h)_ = 8.1, 6.0 MPa) are comparable to those of pristine BIIR-i (*σ*_b(v)_ = 10.2 MPa).

For the composites, the same self-healing mechanism as previously discussed for BIIR-i is assumed.^19,20^ The ionic imidazolium groups of the rubber form a dynamic network which can be healed in case of damage by rearranging ionic clusters. The self-healing process is furthered by the rubber's chain mobility. The silica particles in the composites are assumed to be integrated into the dynamic network *via* interactions of their functional groups with the imidazolium groups. This is reflected in the distinct reinforcing effect discussed above. On the other hand, the particle–matrix interactions seem to suppress the self-healing tendency. This can easily be explained (qualitatively) by a recently suggested modified slip-link model of entangled chains with reversible cross-links in the molten state, which in general favor the self-healing effect.^38^ The presence of additional, slowly moving particles anchored to the polymer will further restrict the mobility of the polymers, resulting in a significant extension of the characteristic self-healing time and thus in a reduced self-healing character.

### Reactive compounding

Comparative studies were made on composites with *in situ* modification of the particle surfaces (C_3–5_). Results concerning dynamic mechanical behavior, tensile properties, and self-healing behavior of these composites in comparison with C_0–2_ are documented in the ESI (SI3–SI7†). The advantage of the *in situ* modification is the saving of a separate processing step (filler functionalization). But it remains unclear to what extent the surface functionalization takes place in this approach. Deviations in the mechanical performance of C_3–5_ in comparison to C_1–2_ indicate that such differences exist. A more detailed exploration of these differences might be the subject of a separate study.

Generally, the tensile properties of C_3–5_ are lower than those of C_1–2_ (see Table 4). Nevertheless, with stress at break values from 9.4 to 11.7 MPa and elongation at break values from 790 to 1030%, the mechanical properties are satisfactory and comparable with those of BIIR-i. In addition, compounds C_4–5_ show a clear reinforcing effect, which manifests in the earlier onset of strain hardening (see ESI SI6†). Although the relaxation behavior and tensile properties of C_3–5_ do not fully match those of C_1–2_ (see ESI SI3–SI6†), the influence of the functional groups on the filler surface as discussed for C_1–2_ is confirmed. Both DMA (see ESI SI3 and SI4†) and tensile tests (see ESI SI5 and SI6†) show the same tendency. Concerning the influence of the filler surface functionality on the reinforcing effect, the following order could be observed: silanol (C_0_) > aliphatic (C_1,4_) > imidazolium (C_2,3,5_). There is a noticeable deviation in the behavior of C_3_ (see ESI SI4 and SI6†), which underlines the uncertainties of reactive compounding.

## Conclusions

The main purpose of the present work was to further improve the inherently very good characteristics of ionically modified bromobutyl rubber (BIIR-i) by compounding with surface-modified silica without sacrificing its excellent self-healing behavior. The compounding was carried out in a reactive process in which the ionic modification of BIIR occurred simultaneously by conversion of the bromine groups with 1-butylimidazole. The interactions between the rubber matrix and silica were adjusted by functional groups on the filler surface. In addition to the naturally occurring silanol groups of silica, alkyl and imidazolium groups were introduced *via* silanization of the particle surface.

The overall performance of silica filled BIIR-i has proven very promising. With tensile strengths of up to 19 MPa and elongation at break values of roughly 1000%, maximum values for a non-covalently cross-linked rubber composite are achieved. These values are significantly better than those of sulfur-crosslinked composites of BIIR with carbon black and layered silicates in which no self-healing occured.^36,37^ The healing efficiencies of the composites (*H*_σ_ = 32–40%, *H*_ε_ = 55–72%) are reduced in comparison to BIIR-i (*H*_σ_ = 87%, *H*_ε_ = 100%), but the absolute tensile stress and elongation at break values of the composites after healing are comparable with those of BIIR-i.

The reinforcing effect caused by the filler could be proven by DMA and stress–strain measurements. In the tensile tests of the composites, an earlier onset of strain hardening and a higher stress-build up at low strain is observed. In the DMA measurements, the reinforcing effect is reflected in an increased storage modulus. The tan *δ* curves of the composites show less pronounced glass transitions indicating reduced chain mobility.

All measurements point to a distinct influence of the filler surface functionalization. Regarding the type of functional groups, the reinforcing effect decreases in the following order: silanol > alkyl > imidazolium. The particularly strong reinforcing effect of pristine silica (S_0_) is attributed to the slightly acidic nature of its silanol groups which undergo strong interactions with the basic imidazolium groups of BIIR-i. For the composites with imidazolium modified particles (C_2–3,5_), a relatively small reinforcing effect was found. Their behavior resembles that of BIIR-i. This is attributed to the similarity of the interacting groups. Both the filler and the matrix of these composites possess imidazolium groups which are assumed to aggregate into ionic clusters which obviously respond similarly to tensile loading as the clusters in BIIR-i. Notably, sample C_2_ shows a significantly better tensile strength than BIIR-i. In the case of composites with alkyl modified particles (C_1,4_), the reinforcing effect is caused by the prevalence of dispersion forces between the nonpolar parts of the rubber backbone and the filler surface which can be pronounced at high surface loading.

The mechanical performance of the composites with *in situ* particle modification (C_3–5_) remains behind that of the composites with pre-modified particle surfaces (C_0–2_). This is attributed to uncomplete conversions on the filler surface. Nevertheless, some improvements in comparison to BIIR-i give rise to the assumption that further improvements might be possible for composites with *in situ* modified particles. This will be the subject of further investigations.

## Conflicts of interest

There are no conflicts to declare.

## Supplementary Material

RA-008-C8RA04631J-s001
