# Supplementary material for: Viscoelastic and self-healing behavior of silica filled ionically modified poly(isobutylene-co-isoprene) rubber
Source: RSC Adv. 2018 Jul 27;8(47):26793–803. doi: 10.1039/c8ra04631j (PMC9083126; doi:10.1039/c8ra04631j)
Supplement: RA-008-C8RA04631J-s001 [file RA-008-C8RA04631J-s001.pdf]

Electronic Supplementary Material (ESI) for RSC Advances.
This journal is © The Royal Society of Chemistry 2018

SUPPORTING INFORMATION

Viscoelastic and self-healing behavior of silica filled ionically modified poly(isobutylene-co-isoprene) rubber

Aladdin Sallat,[1] Amit Das,[1] Jana Schaber,[1] Ulrich Scheler,[1] Eshwaran Subramani Bhagavatheswaran,[1] Klaus Werner Stöckelhuber,[1] Gert Heinrich,[1,3] Brigitte Voit,[1,2] Frank Böhme[1]*

[1] Leibniz Institut für Polymerforschung Dresden, Hohe Straße 6, D-01062 Dresden, Germany

[2] Organische Chemie der Polymere, Technische Universität Dresden, D-01062 Dresden, Germany

[3] Institut für Textilmaschinen und Textile Hochleistungswerkstofftechnik, Technische Universität Dresden, D-01069 Dresden, Germany

Boehme@ipfdd.de

TABLE OF CONTENTS:

**SI1**	TEM micrograph of $\mathbf{C}_0$	2
**SI2**	Picture of test specimens demonstating transparency of $\mathbf{C}_{0\text{-}3}$	2
**SI3**	DMA temperature sweep of $\mathbf{C}_{0\text{-}5}$ and **BIIR-i**	3
**SI4**	DMA temperature strain sweep $\mathbf{C}_{0\text{-}5}$ and **BIIR-i**	4
**SI5**	Mechanical hysteresis curves of composites $\mathbf{C}_{0\text{-}5}$	4
**SI6**	Stress-strain curves of composites $\mathbf{C}_{1\text{-}5}$ and **BIIR-I**	5
**SI7**	Healing tests - Stress-strain curves of **BIIR-I** and composites $\mathbf{C}_{0,2,3}$	5

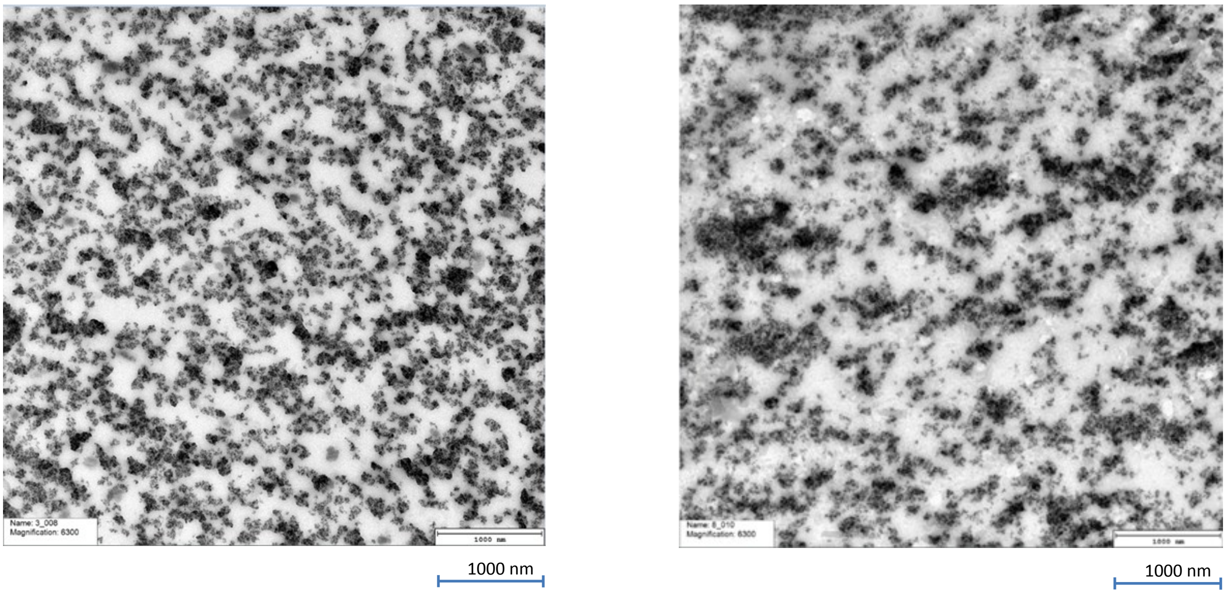

a) b)

SI1 TEM micrographs of a) $\mathbf{C}_0$ and b) $\mathbf{C}_5$. The micrographs were taken with a Libra200 transmission electron microscope (Carl Zeiss Microscopy GmbH, Oberkochen, Germany). For this, approx. 60 nm thin sections of the specimens were prepared using an Ultramicrotome UC6 (Leica Microsystems GmbH, Wetzlar, Germany) at a temperature of -160 °C.

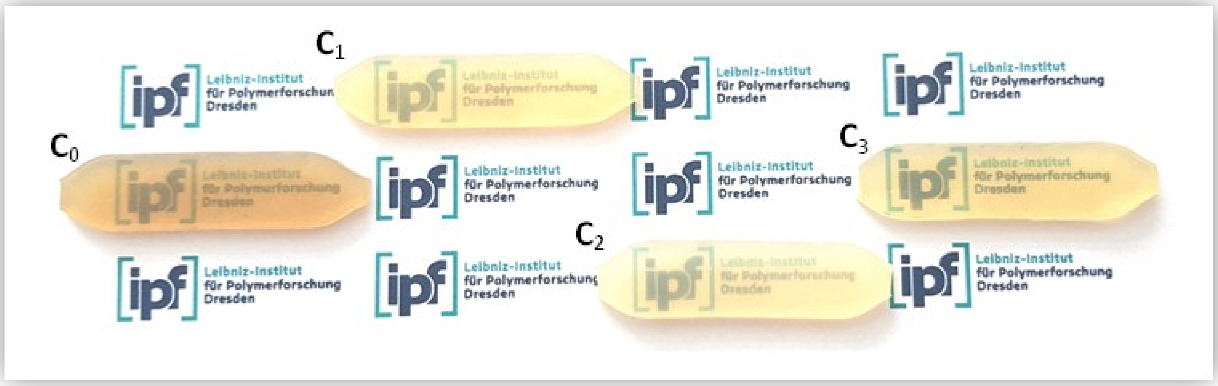

SI2 Picture of test specimens demonstrating transparency of $\mathbf{C}_{0\text{-}3}$

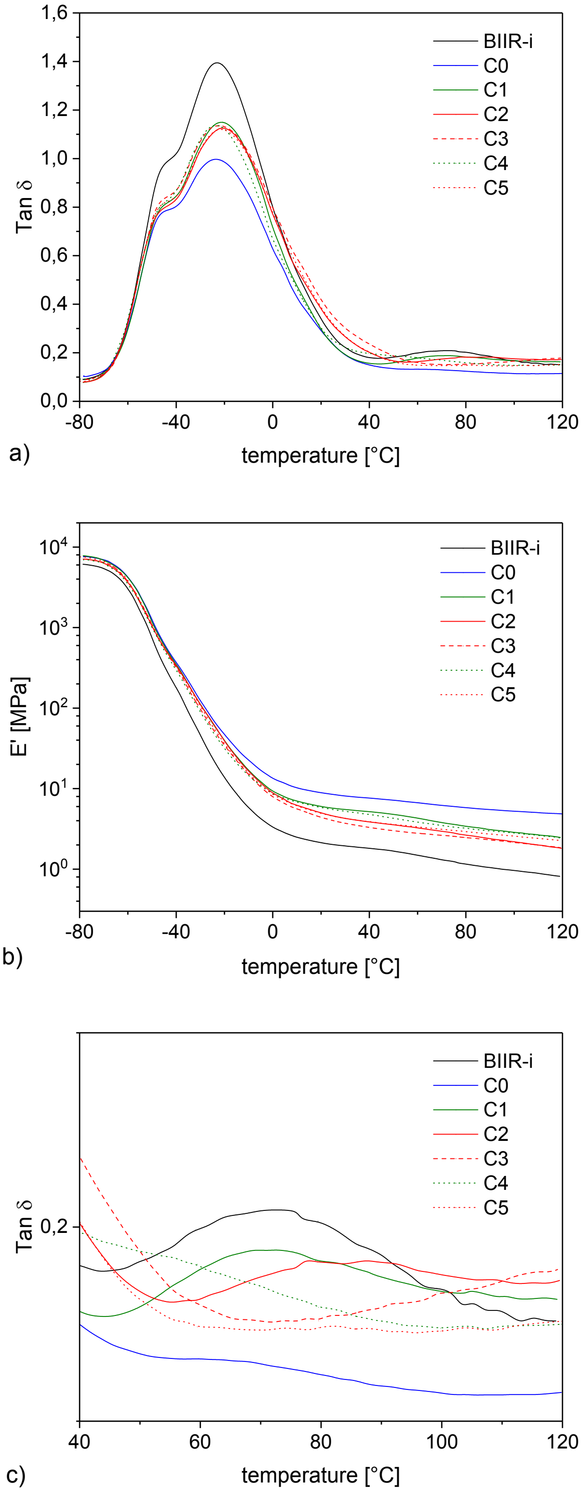

SI3: DMA temperature sweep measurements of rubber-silica composites $\mathbf{C}_{0\text{-}5}$ and **BIIR-i** a) storage modulus plots b) tan δ plots c) enlarged section of the tan δ plots. The curve progressions demonstrate the similarity of the composites with aliphatic ($\mathbf{C}_{1,4}$, green) and ionic ($\mathbf{C}_{2,3,5}$, red) functional groups on the particle surfaces among each other with respect to their temperature dependent dynamic mechanical behavior.

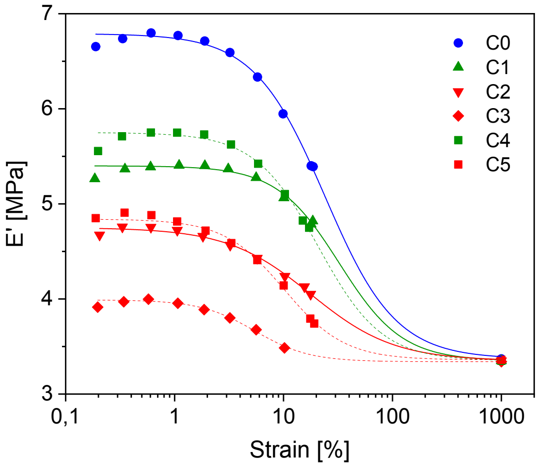

SI4: DMA temperature strain sweep measurements of rubber-silica composites **C**$_{0\text{-}5}$. The curve progression demonstrates the similarity of the composites with aliphatic (**C**$_{1,4}$, green) and ionic (**C**$_{2,3,5}$, red) functional groups on the particle surfaces among each other with respect to their strain dependent dynamic mechanical behavior. The lines are fitted according to the Kraus model. Solid lines represent samples without chemical reactions on the filler surface during compounding. Dotted lines represent samples with chemical reactions on the filler surface during compounding.

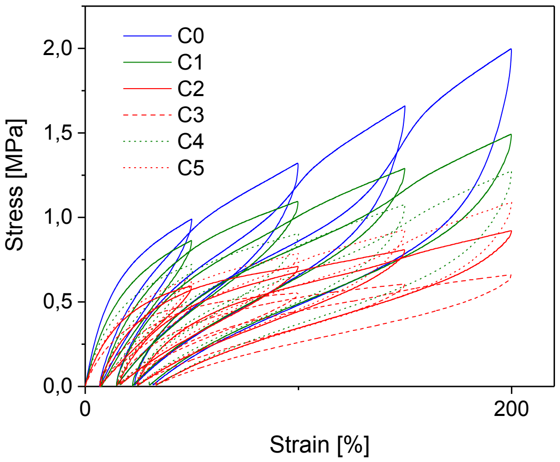

SI5: Mechanical hysteresis curves of compounds **C**$_{0\text{-}5}$. The curve progression demonstrates the similarity of the composites with aliphatic (**C**$_{1,4}$, green) or ionic (**C**$_{2,3,5}$, red) functional groups on the particle surfaces among each other with respect to their stress-strain behavior at low strain.

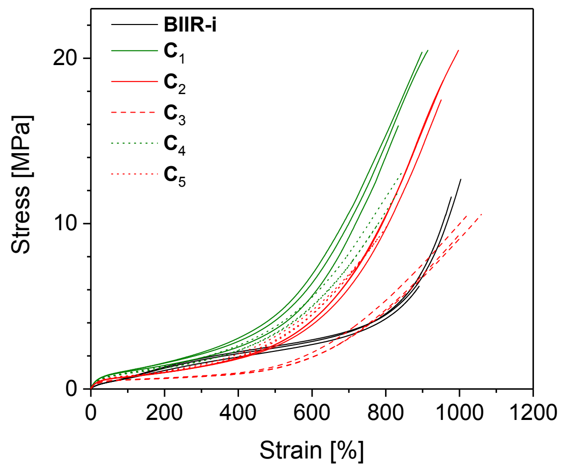

SI6: Stress-strain curves of composites $\mathbf{C}_{1\text{-}5}$ compared to **BIIR-i**

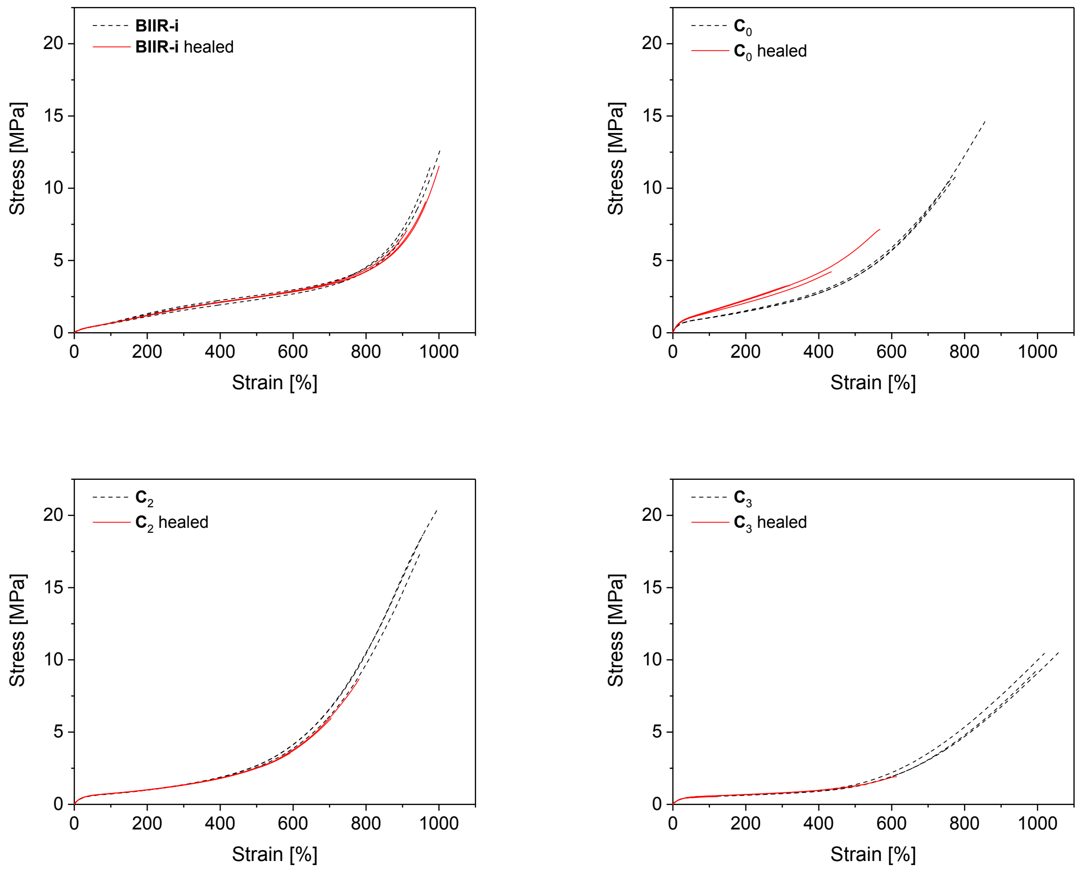

SI7: Healing test. Stress-strain curves of **BIIR-I** and composites $\mathbf{C}_{0,2,3}$. The black (dotted) and the red (solid) curves represent the virgin and the healed samples respectively.
